# 904. Epidemiology of Rotavirus Group A Mixed Genotypes Isolated from Children with Acute Gastroenteritis over a 15-Year Period

**DOI:** 10.1093/ofid/ofad500.949

**Published:** 2023-11-27

**Authors:** Charilaos Dellis, Athanasios Michos, Elizabeth-Barbara Tatsi, Dimitra-Maria koukou, Vasiliki Syriopoulou

**Affiliations:** National and Kapodistrian University of Athens, Athens, Attiki, Greece; First Department of Pediatrics, Infectious Diseases and Chemotherapy Research Laboratory, Medical School, National and Kapodistrian University of Athens, ‘Aghia Sophia’ Children’s Hospital, Athens, Greece, Athens, Attiki, Greece; University Research Institute for Maternal and Child Health and Precision Medicine, Athens, Greece, Athens, Attiki, Greece; National and Kapodistrian University of Athens, Athens, Attiki, Greece; First Department of Pediatrics, Infectious Diseases and Chemotherapy Research Laboratory, Medical School, National and Kapodistrian University of Athens, ‘Aghia Sophia’ Children’s Hospital, Athens, Greece, Athens, Attiki, Greece

## Abstract

**Background:**

Limited data exist worldwide regarding the genetic epidemiology of mixed G and/or P Rotavirus group A (RVA) strains and their association with demographic and clinical characteristics in children with acute gastroenteritis (AGE).

**Methods:**

Positive fecal samples for mixed RVA previously G (VP7) and P (VP4) genotyped from children aged ≤16 years with AGE between 2007-2021 (15 years) in Greece were further genotyped for I (VP6) and E (NSP4) using RT-PCR and Sanger sequencing. Demographic, clinical and laboratory data of children infected with a mixed RVA strain were also collected.

**Results:**

Over the 15-year period, 129/4427 (2.9%) mixed RVA strains were detected. Higher detection rate was observed in 2016 (7.5%) and in 2021 (5.8%) **(Figure 1)**. Children infected with a mixed RVA strain had a median age of 21.1 months (IQR: 56.2) and were predominantly males (85/129, 65.9%). Mixed G genotype were 73/129 (56.6%), mixed P type 54/129 (41.1%) and both G and P mixed genotypes 3/129 (2.3%). In total, 27 different G/P combinations were detected with most prevalent the: G1G4P[8] (47/129, 36.4%), G1P[4]P[8] (18/129, 14%) and G4P[4]P[8] (18/129, 14%) **(Figure 2)**. Higher seasonal peaks were observed for G mixed types in spring (68.8%) and for P mixed types in summer (66.7%). Infection with a G mixed type had a higher relative frequency in children aged 0-12 months, while infection with P mixed type had a higher relative frequency in children aged 13-48 months (*p*=0.043). I and E typing was successful in 49.6% (64/129) of mixed samples from 2013-2021. Two different I (I1, I2) and three different E (E1, E2, E3) genotypes were detected, with I1 (42.2%, 27/64) and E1 (39.1%, 25/64) being the most common I and E genotypes, respectively. In 31.3% (20/64) of samples, mixed I (I1I1, I1I2) and/or E (E1E1, E1E2) genotypes were also detected. No association has been detected between mixed genotypes and clinical characteristics of the patients.

Annual distribution of total and mixed RVA cases in a 15-year period

**Figure:**
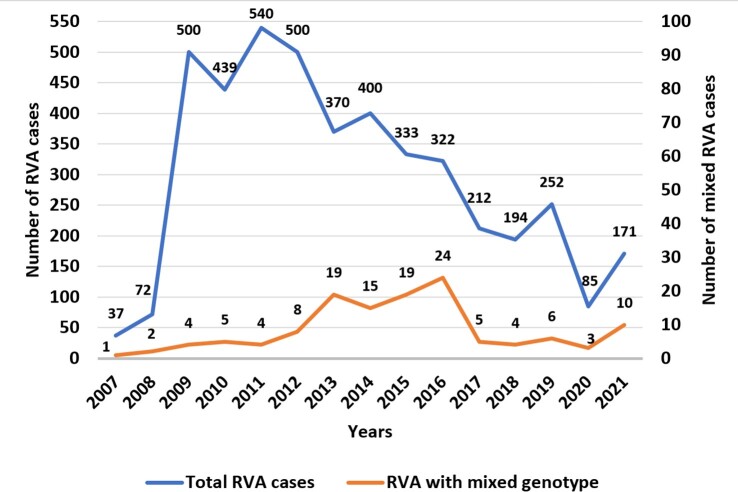

G-P combinations of RVA mixed strains.

**Figure:**
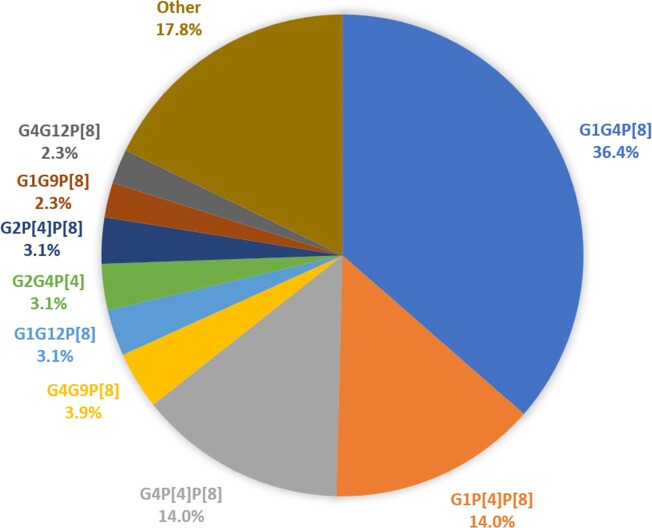

Other combinations found in a smaller proportion (<2.3%) were: G3P[4]P[8] (1.6%), G4P[8]P[9] (1.6%), G6P[4]P[9] (1.6%), GUDP[4]P[8] (1.6%), G2G4P[4]P[8] (1.6%), G1G2P[4] (0.8 %), G1G3P[9] (0.8%), G1G8P[4]P[8] (0.8%), G10P[8]P[10] (0.8%), G12P[4]P[8] (0.8%), G2P[8]P[10] (0.8%), G2G4P[8] (0.8%), G2G9P[8] (0.8%), G4G12P[4] (0.8%), G8G9P[8] (0.8%), G9P[4]P[8] (0.8%), G9P[6]P[8] (0.8%), G9G12P[8](0.8%).

**Conclusion:**

These results indicate the great genetic diversity of RVA strains. Significant differences were found in the seasonality of the mixed G and P strains as well as in the age group of children infected with mixed G and P strains.

**Disclosures:**

**All Authors**: No reported disclosures

